# Development of a multivariable prediction model for severe COVID-19 disease: a population-based study from Hong Kong

**DOI:** 10.1038/s41746-021-00433-4

**Published:** 2021-04-08

**Authors:** Jiandong Zhou, Sharen Lee, Xiansong Wang, Yi Li, William Ka Kei Wu, Tong Liu, Zhidong Cao, Daniel Dajun Zeng, Keith Sai Kit Leung, Abraham Ka Chung Wai, Ian Chi Kei Wong, Bernard Man Yung Cheung, Qingpeng Zhang, Gary Tse

**Affiliations:** 1 School of Data Science, City University of Hong Kong, Hong Kong, China; 2 Cardiovascular Analytics Group, Laboratory of Cardiovascular Physiology, Hong Kong, China; 3 Li Ka Shing Institute of Health Sciences, Hong Kong, China; 4 Department of Cardiothoracic Surgery, Wuhan Asia Heart Hospital Affiliated to Wuhan University of Science and Technology, Hubei, Wuhan, China; 5 Tianjin Key Laboratory of Ionic-Molecular Function of Cardiovascular Disease, Department of Cardiology, Tianjin Institute of Cardiology, Second Hospital of Tianjin Medical University, Tianjin, China; 6 Institute of Automation, Chinese Academy of Sciences, Beijing, China; 7 Emergency Medicine Unit, LKS Faculty of Medicine, University of Hong Kong, Pokfulam, Hong Kong, China; 8 Department of Pharmacology and Pharmacy, University of Hong Kong, Pokfulam, Hong Kong, China; 9 Medicines Optimisation Research and Education (CMORE), UCL School of Pharmacy, London, United Kingdom; 10 Department of Medicine, University of Hong Kong, Pokfulam, Hong Kong, China

**Keywords:** Risk factors, Medical research

## Abstract

Recent studies have reported numerous predictors for adverse outcomes in COVID-19 disease. However, there have been few simple clinical risk scores available for prompt risk stratification. The objective is to develop a simple risk score for predicting severe COVID-19 disease using territory-wide data based on simple clinical and laboratory variables. Consecutive patients admitted to Hong Kong’s public hospitals between 1 January and 22 August 2020 and diagnosed with COVID-19, as confirmed by RT-PCR, were included. The primary outcome was composite intensive care unit admission, need for intubation or death with follow-up until 8 September 2020. An external independent cohort from Wuhan was used for model validation. COVID-19 testing was performed in 237,493 patients and 4442 patients (median age 44.8 years old, 95% confidence interval (CI): [28.9, 60.8]); 50% males) were tested positive. Of these, 209 patients (4.8%) met the primary outcome. A risk score including the following components was derived from Cox regression: gender, age, diabetes mellitus, hypertension, atrial fibrillation, heart failure, ischemic heart disease, peripheral vascular disease, stroke, dementia, liver diseases, gastrointestinal bleeding, cancer, increases in neutrophil count, potassium, urea, creatinine, aspartate transaminase, alanine transaminase, bilirubin, D-dimer, high sensitive troponin-I, lactate dehydrogenase, activated partial thromboplastin time, prothrombin time, and C-reactive protein, as well as decreases in lymphocyte count, platelet, hematocrit, albumin, sodium, low-density lipoprotein, high-density lipoprotein, cholesterol, glucose, and base excess. The model based on test results taken on the day of admission demonstrated an excellent predictive value. Incorporation of test results on successive time points did not further improve risk prediction. The derived score system was evaluated with out-of-sample five-cross-validation (AUC: 0.86, 95% CI: 0.82–0.91) and external validation (*N* = 202, AUC: 0.89, 95% CI: 0.85–0.93). A simple clinical score accurately predicted severe COVID-19 disease, even without including symptoms, blood pressure or oxygen status on presentation, or chest radiograph results.

## Introduction

The coronavirus disease 2019 has a wide clinical spectrum, with disease severities ranging from completely asymptomatic to the need for intubation and death^1–4^. For example, those with existing cardiac problems are more likely to suffer from more severe disease life courses^5–11^, with potential modifier effects from different medication classes^12–14^. Aside from comorbidities, numerous risk factors such as high D-dimer^15^, neutrophil^16^, and liver damage^17^ and deranged clotting^18^ have been associated with disease severity. Such patients may benefit from early aggressive treatment^19–23^. However, to date, there are only a few easy-for-use risk models that can be used for early identification of such at-risk individuals in clinical practice^24,25^. The aim of the study is to extend these previous findings and develop a predictive risk score based on demographic, comorbidity, medication record, and laboratory data using territory-wide electronic health records, without clinical parameters or imaging results. We hypothesized that incorporation of test results on successive time points would improve risk prediction. The model was validated internally, and externally using a single-center cohort from Wuhan.

## Results

### Basic characteristics

A total of 4442 patients (median age 44.8 years old, 95% CI: [28.9, 60.8]); 50% males) were diagnosed with the COVID-19 infection between 1 January 2020 and 22 August 2020 in Hong Kong public hospitals or their associated ambulatory/outpatient facilities (Table 1). On follow-up until 8 September 2020, a total of 212 patients (4.77%) met the primary outcome of need for intensive care admission or intubation, or death. The survival curve is presented in Fig. 1. The sudden inflexion point at 200 days likely reflects the surge of new cases around this period. The baseline and clinical characteristics of male and female COVID-19 patients are provided in Supplementary Table 3.

**Table 1:** Baseline clinical characteristics of patients with COVID-19.

Characteristics	Overall (*N* = 4442); median (IQR); max; *N* or count (%)	Composite outcome (*N* = 209) median (IQR); max; *N* or count (%)	No. of composite outcome (*N* = 4233); median (IQR); max; *N* or count (%)	*P* value^#^
**Outcomes**				
Composite	209 (4.70%)	209 (100.00%)	0 (0.00%)	<0.0001***
Mortality	93 (2.09%)	93 (44.49%)	0 (0.00%)	<0.0001***
ICU	96 (2.16%)	96 (45.93%)	0 (0.00%)	<0.0001***
Intubation	98 (2.20%)	98 (46.88%)	0 (0.00%)	<0.0001***
**Demographics**				
Male gender	2227 (50.13%)	138 (66.02%)	2089 (49.35%)	0.0115*
Age	44.8 (28.9–60.7); 100.6; *n* = 4442	70.99 (61.8–82.6); 98.7; *n* = 209	43.1 (28.1–59.3); 100.6; *n* = 4233	<0.0001***
[60, 64]	401 (9.02%)	29 (13.87%)	372 (8.78%)	0.0339*
[65, 69]	289 (6.50%)	29 (13.87%)	260 (6.14%)	0.0001***
[70, 74]	194 (4.36%)	25 (11.96%)	169 (3.99%)	<0.0001***
≥75	282 (6.34%)	85 (40.66%)	197 (4.65%)	<0.0001***
**Comorbidities**				
Diabetes mellitus	74 (1.66%)	18 (8.61%)	56 (1.32%)	<0.0001***
Hypertension	601 (13.52%)	107 (51.19%)	494 (11.67%)	<0.0001***
Heart failure	5 (0.11%)	3 (1.43%)	2 (0.04%)	<0.0001***
Atrial fibrillation	43 (0.96%)	10 (4.78%)	33 (0.77%)	<0.0001***
Liver diseases	7 (0.15%)	2 (0.95%)	5 (0.11%)	0.0376*
Dementia and Alzheimer	8 (0.18%)	4 (1.91%)	4 (0.09%)	<0.0001***
COPD	34 (0.76%)	3 (1.43%)	31 (0.73%)	0.4709
Ischemic heart disease	110 (2.47%)	23 (11.00%)	87 (2.05%)	<0.0001***
Peripheral vascular disease	7 (0.15%)	3 (1.43%)	4 (0.09%)	0.0001***
Stroke	70 (1.57%)	21 (10.04%)	49 (1.15%)	<0.0001***
Gastrointestinal bleeding	71 (1.59%)	14 (6.69%)	57 (1.34%)	<0.0001***
Cancer	95 (2.13%)	20 (9.56%)	75 (1.77%)	<0.0001***
Obesity	6 (0.13%)	1 (0.47%)	5 (0.11%)	0.6763
**Medications**				
ACEI	160 (3.60%)	35 (16.74%)	125 (2.95%)	<0.0001***
ARB	149 (3.35%)	23 (11.00%)	126 (2.97%)	<0.0001***
Steroid	258 (5.80%)	17 (8.13%)	241 (5.69%)	0.2204
Lopinavir/ritonavir	671 (15.10%)	54 (25.83%)	617 (14.57%)	0.0004***
Ribavirin	527 (11.86%)	30 (14.35%)	497 (11.74%)	0.3713
Interferon beta-1B	716 (16.11%)	68 (32.53%)	648 (15.30%)	<0.0001***
Hydroxychloroquine	28 (0.63%)	3 (1.43%)	25 (0.59%)	0.2959
Calcium channel blockers	477 (10.73%)	72 (34.44%)	405 (9.56%)	<0.0001***
Beta blockers	205 (4.61%)	40 (19.13%)	165 (3.89%)	<0.0001***
Diuretics for hypertension	54 (1.21%)	9 (4.30%)	45 (1.06%)	0.0002***
Nitrates	62 (1.39%)	13 (6.22%)	49 (1.15%)	<0.0001***
Antihypertensive drugs	92 (2.07%)	21 (10.04%)	71 (1.67%)	<0.0001***
Antidiabetic drugs	236 (5.31%)	64 (30.62%)	172 (4.06%)	<0.0001***
Statins and fibrates	390 (8.77%)	68 (32.53%)	322 (7.60%)	<0.0001***
Lipid-lowering drugs	379 (8.53%)	66 (31.57%)	313 (7.39%)	<0.0001***
Anticoagulants	157 (3.53%)	67 (32.05%)	90 (2.12%)	<0.0001***
Antiplatelets	190 (4.27%)	40 (19.13%)	150 (3.54%)	<0.0001***
**Complete blood count**				
Mean corpuscular volume, fL	86.8 (82.9–90.12);110.6; *n* = 2391	89.0 (85.1–92.6); 105.9; *n* = 140	86.7 (82.84–90.0); 110.6; *n* = 2251	<0.0001***
Basophil, ×10^9^/L	0.01 (0.0–0.02); 0.2; *n* = 2919	0.0 (0.0–0.01); 0.13; *n* = 142	0.01 (0.0–0.02); 0.2; *n* = 2777	0.0004***
Eosinophil, ×10^9^/L	0.03 (0.0–0.1); 3.53; *n* = 3037	0.0 (0.0–0.02); 0.96; *n* = 157	0.03 (0.0–0.1); 3.53; *n* = 2880	<0.0001***
Lymphocyte, ×10^9^/L	1.35 (0.98–1.82); 16.99; *n* = 3045	0.86 (0.6–1.2); 3.09; *n* = 157	1.39 (1.0–1.85); 16.99; *n* = 2888	<0.0001***
Metamyelocyte, ×10^9^/L	0.1 (0.07–0.21); 0.7; *n* = 14	0.19 (0.08–0.28); 0.7; *n* = 7	0.09 (0.06–0.12); 0.23; *n* = 7	0.2003
Monocyte, ×10^9^/L	0.49 (0.36–0.61); 3.15; *n* = 3045	0.48 (0.33–0.7); 1.67; *n* = 157	0.49 (0.36–0.6); 3.15; *n* = 2888	0.7101
Neutrophil, ×10^9^/L	3.23 (2.4–4.39); 18.63; *n* = 3045	4.64 (3.49–6.2); 18.63; *n* = 157	3.2 (2.36–4.3); 16.337; *n* = 2888	<0.0001***
White blood count, ×10^9^/L	5.34 (4.3–6.72); 23.9; *n* = 3102	6.1 (4.72–8.31); 21.19; *n* = 159	5.3 (4.24–6.69); 23.9; *n* = 2943	<0.0001***
Mean cell hemoglobin, pg	29.9 (28.5–31.3); 37.0; *n* = 3102	31.3 (29.35–33.2); 36.2; *n* = 159	29.9 (28.4–31.1); 37.0; *n* = 2943	<0.0001***
Myelocyte, ×10^9^/L	0.22 (0.07–0.36); 1.29; *n* = 30	0.35 (0.2–0.41); 1.29; *n* = 15	0.08 (0.06–0.22); 0.41; *n* = 15	0.0127*
Platelet, ×10^9^/L	215.0 (174.0–269.0); 778.0; *n* = 3102	176.0 (141.0–216.5); 778.0; *n* = 159	217.0 (176.0–271.0); 722.0; *n* = 2943	<0.0001***
Reticulocyte, ×10^9^/L	41.3 (29.7–74.2); 318.0; *n* = 12	57.0 (57.0–57.0); 57.0; *n* = 1	39.93 (29.7–74.19); 318.0; *n* = 11	0.7721
Red blood count, ×10^12^/L	4.67 (4.32–5.07); 7.45; *n* = 3103	4.46 (3.92–4.82); 7.27; *n* = 159	4.68 (4.33–5.08); 7.45; *n* = 2944	<0.0001***
Hematocrit, L/L	0.4 (0.37–0.43); 0.516; *n* = 508	0.38 (0.35–0.42); 0.504; *n* = 23	0.4 (0.37–0.43); 0.516; *n* = 485	0.0549
**Liver and renal function tests**				
K/potassium, mmol/L	3.8 (3.57–4.1); 7.7; *n* = 2289	3.8 (3.5–4.1);7.7; *n* = 142	3.8 (3.58–4.1); 6.96; *n* = 2147	0.4534
Urate, mmol/L	0.3 (0.2–0.4); 0.635; *n* = 51	0.29 (0.19–0.32); 0.62; *n* = 7	0.29 (0.23–0.39); 0.635; *n* = 44	0.5111
Albumin, g/L	41.0 (37.0–44.0); 201.0; *n* = 2302	36.0 (30.0–39.0); 48.3; *n* = 144	41.0 (37.71–44.2); 201.0; *n* = 2158	<0.0001***
Na/sodium, mmol/L	139.0 (137.0–140.6); 147.1; *n* = 2295	136.5 (133.0–139.3); 147.0; *n* = 142	139.0 (137.0–140.7); 147.1; *n* = 2153	<0.0001***
Urea, mmol/L	3.9 (3.1–4.88); 59.3; *n* = 2294	6.0 (4.3–7.98); 59.3; *n* = 142	3.86 (3.1–4.74); 31.64; *n* = 2152	<0.0001***
Protein, g/L	74.0 (70.2–77.72); 92.7; *n* = 2034	70.0 (66.0–75.5); 86.0; *n* = 121	74.0 (70.7–77.9); 92.7; *n* = 1913	<0.0001***
Creatinine, μmol/L	70.0 (58.1–84.0); 1280.0; *n* = 2304	86.95 (70.5–111.0); 1280.0; *n* = 144	69.0 (58.0–83.0); 834.0; *n* = 2160	<0.0001***
Alkaline phosphatase, U/L	66.0 (54.0–81.0); 550.0; *n* = 2292	72.0 (56.0–91.0); 275.9; *n* = 141	65.8 (54.0–81.0); 550.0; *n* = 2151	0.0131*
Aspartate transaminase, U/L	27.0 (21.0–41.55); 1713.0; *n* = 644	44.0 (28.0–65.5); 1713.0; *n* = 55	26.0 (21.0–39.0); 863.0; *n* = 589	<0.0001***
Alanine transaminase, U/L	23.0 (16.0–35.0); 902.0; *n* = 1818	32.15 (20.0–53.5); 902.0; *n* = 108	23.0 (16.0–34.0); 320.0; *n* = 1710	<0.0001***
Bilirubin, μmol/L	7.5 (5.4–10.5); 148.4; *n* = 2291	9.0 (6.3–13.0); 148.4; *n* = 141	7.3 (5.2–10.2); 109.0; *n* = 2150	<0.0001***
**Lipid and glucose tests**				
Triglyceride, mmol/L	1.4 (1.0–2.0); 9.35; *n* = 290	1.52 (1.01–2.02); 5.67; *n* = 52	1.4 (1.0–2.0); 9.35; *n* = 238	0.6893
Low-density lipoprotein, mmol/L	2.5 (1.9–3.1); 6.9; *n* = 259	1.9 (1.5–2.5); 5.0; *n* = 41	2.5 (2.04–3.3); 6.9; *n* = 218	<0.0001***
High-density lipoprotein, mmol/L	1.1 (0.87–1.3); 2.97; *n* = 268	0.9 (0.7–1.3); 1.86; *n* = 43	1.1 (0.9–1.3); 2.97; *n* = 225	0.0307*
Cholesterol, mmol/L	4.34 (3.67–5.1); 9.43; *n* = 272	3.8 (2.76–4.65); 6.97; *n* = 44	4.46 (3.8–5.2); 9.43; *n* = 228	0.0002***
Clearance, mL/min	107.3 (90.2–111.4); 115.6; *n* = 3	94.34 (94.34–94.3); 115.6285; *n* = 2	107.26 (107.26–107.26); 107.2595; *n* = 1	0.5403
HbA1c, g/dL	13.6 (12.6–14.7); 94.1; *n* = 3117	13.3 (11.8–14.55); 81.4; *n* = 162	13.7 (12.7–14.7); 94.1; *n* = 2955	0.0069**
Glucose, mmol/L	5.67 (5.06–6.89); 32.1; *n* = 2203	6.95 (5.8–9.1); 18.6; *n* = 139	5.6 (5.03–6.7); 32.1; *n* = 2064	<0.0001***
**Cardiac, clotting, inflammatory, and acid–base tests**				
D-dimer, ng/mL	296.7 (156.5–555.1); 10,000.0; *n* = 588	834.0 (393.9–1252.6); 10,000.0; *n* = 55	280.0 (149.9–473.0); 6579.9; *n* = 533	<0.0001***
High sensitive troponin-I, ng/L	3.0 (1.5–6.37); 12,827.6; *n* = 1386	10.0 (4.77–37.12);12,827.6; *n* = 90	3.0 (1.4–5.58); 1598.7; *n* = 1296	<0.0001***
Lactate dehydrogenase, U/L	196.0 (166.0–244.0); 1116.0; *n* = 2472	320.0 (229.5–437.5); 1116.0; *n* = 131	194.0 (164.5–235.4); 969.8; *n* = 2341	<0.0001***
APTT, second	30.6 (27.5–34.2); 120.0; *n* = 1593	32.35 (28.15–35.55); 120.0; *n* = 144	30.5 (27.5–34.1); 55.2; *n* = 1449	0.0132*
Prothrombin time/INR, second	12.0 (11.4–12.6); 110.0; *n* = 1110	12.4 (11.7–13.2); 110.0; *n* = 100	12.0 (11.4–12.6); 43.4; *n* = 1010	0.0001***
C-reactive protein, mg/dL	0.36 (0.13–1.4); 33.99; *n* = 3108	5.07 (1.61–10.22); 32.529; *n* = 165	0.32 (0.12–1.19); 33.99; *n* = 2943	<0.0001***
HCO_3_/bicarbonate, mmol/L	23.0 (20.0–25.05); 32.8; *n* = 166	22.7 (20.5–25.2); 32.8; *n* = 67	23.2 (19.95–25.0); 31.0; *n* = 99	0.7696
Base excess, mmol/L	−0.5 (−2.5 to 1.5); 9.5; *n* = 510	−1.4 (−3.7 to 0.5); 6.4; *n* = 178	−0.05(−1.8 to 1.9); 9.5; *n* = 332	<0.0001***
Blood pCO_2_, kPa	4.89 (4.15–5.74); 10.94; *n* = 511	4.61 (3.92–5.51); 10.91; *n* = 178	5.07 (4.28–5.84); 10.94; *n* = 333	0.0011**
Blood pH	7.42 (7.38–7.47); 7.612; *n* = 511	7.43 (7.37–7.47); 7.612; *n* = 178	7.42 (7.38–7.47); 7.6; *n* = 333	0.8895
Calcium, mmol/L	1.13 (1.09–1.17); 1.33; *n* = 36	1.13 (1.07–1.19); 1.33; *n* = 25	1.14 (1.12–1.16); 1.31; *n* = 11	0.5472

*COPD* chronic obstructive pulmonary disease, *ACEI* angiotensinogen-converting enzyme inhibitor, *ARB* angiotensin receptor blocker, *APTT* activated partial thromboplastin time.

*p ≤ 0.05, **p ≤ 0.01, ***p ≤ 0.001.

# indicates that the comparisons were made between patients meeting primary outcome vs. those that did not.

**Fig. 1:**
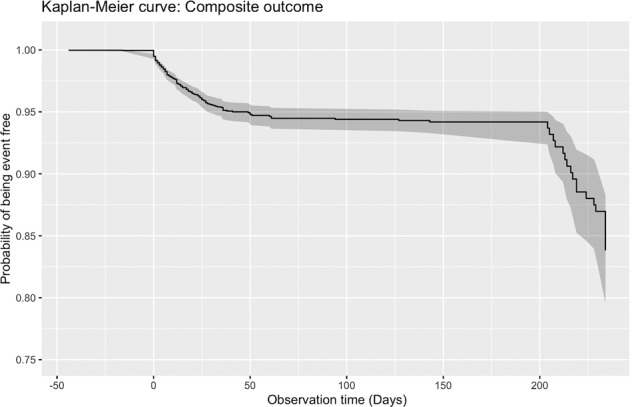
Survival analysis for the cohort from Hong Kong, China. Survival curve of COVID-19 patients for the primary outcome, a composite of intensive care admission, need for intubation or death.

### Development of a clinical risk score and validation

Univariate logistic regression analyses are shown in Table 2, which identified the significant risk predictors for the composite outcome. However, for clinical practice, it is impractical to precisely input the values of all variables assessed from the different domains of the health records. Three different models were developed (Tables 3–5), as detailed in the “Methods” section. The easy-to-use score system is shown in Table 6.

**Table 2:** Univariate analysis of significant risk factors to predict severe COVID-19 disease.

Characteristics	Beta coefficient	Cut-off	HR (95% CI for HR)	Wald test	*P* value
**Demographics**					
Male gender	0.65	–	1.92 (1.44–2.56)	20	<0.0001***
Age	0.07	–	1.08 (1.07–1.09)	300	<0.0001***
[60, 64]	0.46	–	1.59 (1.07–2.35)	5.3	0.021*
[65, 69]	0.89	–	2.44 (1.64–3.61)	20	<0.0001***
[70, 74]	0.97	–	2.63 (1.73–4.01)	20	<0.0001***
≥75	2.3	–	10.1 (7.62–13.3)	260	<0.0001***
**Past comorbidities**					
Diabetes mellitus	1.7	–	5.38 (3.31–8.74)	46	<0.0001***
Hypertension	2	–	7.12 (5.42–9.36)	200	<0.0001***
Heart failure	0.78	–	2.18 (0.31–15.6)	0.6	0.44
Atrial fibrillation	1.7	–	5.56 (2.94–10.5)	28	<0.0001***
Liver diseases	1.7	–	5.52 (1.37–22.3)	5.8	0.017*
Dementia and Alzheimer	2.3	–	9.83 (3.64–26.5)	20	<0.0001***
COPD	0.87	–	2.38 (0.76–7.45)	2.2	0.14
Ischemic heart disease	1.6	–	4.74 (3.07–7.32)	49	<0.0001***
Peripheral vascular disease	2.7	–	15.2 (4.84–47.5)	22	<0.0001***
Stroke	2	–	7.22 (4.59–11.3)	73	<0.0001***
Gastrointestinal bleeding	1.6	–	4.82 (2.8–8.29)	32	<0.0001***
Cancer	1.8	–	5.88 (3.7–9.33)	56	<0.0001***
Obesity	1.5	–	4.64 (0.65–33.2)	2.4	0.13
**Medications**					
ACEI	1.6	–	4.85 (3.35–7.03)	70	<0.0001***
ARB	1.1	–	2.97 (1.91–4.61)	23	<0.0001***
Steroid	0.35	–	1.41 (0.86–2.32)	1.9	0.17
Lopinavir/ritonavir	0.62	–	1.86 (1.35–2.55)	15	0.0001***
Ribavirin	0.16	–	1.17 (0.793–1.74)	0.64	0.42
Interferon beta-1B	1.1	–	2.95 (2.19–3.97)	51	<0.0001***
Hydroxychloroquine	0.71	–	2.03 (0.65–6.37)	1.5	0.22
Calcium channel blockers	1.4	–	3.92 (2.93–5.24)	85	<0.0001***
Beta blockers	1.5	–	4.46 (3.15–6.32)	71	<0.0001***
Diuretics for hypertension	1.2	–	3.33 (1.71–6.5)	12	0.00042***
Nitrates	1.2	–	3.45 (1.95–6.11)	18	<0.0001***
Antihypertensive drugs	1.4	–	4.1 (2.59–6.51)	36	<0.0001***
Antidiabetic drugs	1.9	–	6.75 (4.97–9.16)	150	<0.0001***
Statins and fibrates	1.5	–	4.57 (3.41–6.13)	100	<0.0001***
Lipid-lowering drugs	1.5	–	4.51 (3.35–6.07)	99	<0.0001***
Anticoagulants	2.4	–	10.9 (7.97–14.8)	230	<0.0001***
Antiplatelets	1.5	–	4.61 (3.25–6.54)	73	<0.0001***
**Complete blood count**					
Mean corpuscular volume, fL	0.046	83.4	1.05 (1.02–1.07)	12	0.0004***
Basophil, ×10^9^/L	−11	0.03	1.41e−05 (9.93e−10–0.202)	5.2	0.022*
Eosinophil, ×10^9^/L	−4.5	0.058	0.012 (0.001–0.141)	12	0.0005***
Lymphocyte, ×10^9^/L	−1.5	1.48	0.223 (0.159–0.313)	75	<0.0001***
Metamyelocyte, ×10^9^/L	0.52	0.23	1.68 (0.0359–78.7)	0.07	0.79
Monocyte, ×10^9^/L	0.62	0.38	1.86 (1.03–3.36)	4.3	0.038*
Neutrophil, ×10^9^/L	0.28	5.43	1.32 (1.27–1.38)	180	<0.0001***
White blood count, ×10^9^/L	0.19	7.54	1.21 (1.16–1.26)	78	<0.0001***
Mean cell hemoglobin, pg	0.18	32.6	1.19 (1.12–1.27)	31	<0.0001***
Myelocyte, ×10^9^/L	3	0.41	20.5 (2.7–155)	8.5	0.0035**
Platelet, ×10^9^/L	−0.0083	321	0.992 (0.989–0.994)	38	<0.0001***
Reticulocyte, ×10^9^/L	−0.0022	42.6	0.998 (0.967–1.03)	0.02	0.89
Red blood count, ×10^12^/L	−0.69	4.75	0.501 (0.391–0.642)	30	<0.0001***
Hematocrit, L/L	−9	0.38	0.000125 (3.35e−08–0.464)	4.6	0.032*
**Liver and renal function tests**					
K/potassium, mmol/L	0.39	4.35	1.48 (1.02–2.14)	4.4	0.037*
Urate, mmol/L	−2.5	0.17	0.0812 (0.0001–51.7)	0.58	0.45
Albumin, g/L	−0.096	33.8	0.909 (0.896–0.921)	180	<0.0001***
Na/sodium, mmol/L	−0.18	135.24	0.836 (0.808–0.864)	110	<0.0001***
Urea, mmol/L	0.088	6.1	1.09 (1.08–1.11)	160	<0.0001***
Protein, g/L	−0.037	67	0.963 (0.95–0.976)	30	<0.0001***
Creatinine, μmol/L	0.0033	97.2	1.0033 (1.003–1.004)	86	<0.0001***
Alkaline phosphatase, U/L	0.0012	94.6	1.0012 (0.998-1.005)	0.56	0.45
Aspartate transaminase, U/L	0.002	41.8	1.002 (1.001–1.003)	17	<0.0001***
Alanine transaminase, U/L	0.0067	25.8	1.01 (1–1.01)	48	<0.0001***
Bilirubin, μmol/L	0.028	10.8	1.03 (1.02–1.04)	26	<0.0001***
**Lipid and glucose tests**					
Triglyceride, mmol/L	−0.031	2.07	0.969 (0.759–1.24)	0.06	0.8
Low-density lipoprotein, mmol/L	−0.74	1.78	0.476 (0.321–0.706)	14	0.0002***
High-density lipoprotein, mmol/L	−1.3	0.9	0.28 (0.102–0.769)	6.1	0.013*
Cholesterol, mmol/L	−0.6	3.1	0.548 (0.406–0.74)	15	<0.0001***
HbA1c, g/dL	0.015	13.95	1.02 (0.997–1.03)	2.6	0.11
Glucose, mmol/L	0.12	5.55	1.13 (1.09–1.17)	44	<0.0001***
**Cardiac, clotting, inflammatory, and acid–base tests**					
D-dimer, ng/mL	0.0005	831.46	1.0004 (1.0003–1.0006)	50	<0.0001***
High sensitive troponin-I, ng/L	0.0003	9.95	1.0003 (1.0002–1.0004)	17	<0.0001***
Lactate dehydrogenase, U/L	0.0056	289	1.006 (1.005–1.006)	230	<0.0001***
APTT, second	0.031	32.8	1.03 (1.02–1.05)	18	<0.0001***
Prothrombin time/INR, second	0.024	13	1.02 (1.01–1.04)	8.1	0.0044**
C-reactive protein, mg/dL	0.14	0.88	1.15 (1.13–1.17)	290	<0.0001***
HCO_3_/bicarbonate, mmol/L	−0.014	23.5	0.986 (0.928–1.05)	0.19	0.67
Base excess, mmol/L	−0.096	−3.5	0.908 (0.876–0.942)	26	<0.0001***
Blood pCO_2_, kPa	−0.11	3.62	0.898 (0.793–1.02)	2.9	0.09
Blood pH	−1.7	7.49	0.178 (0.027–1.18)	3.2	0.073
Calcium, mmol/L	−1.6	0.97	0.195 (0.0028–13.4)	0.57	0.45

*COPD* chronic obstructive pulmonary disease, *ACEI* angiotensinogen-converting enzyme inhibitor, *ARB* angiotensin receptor blocker, *APTT* activated partial thromboplastin time.

*p ≤ 0.05, **p ≤ 0.01, ***p ≤ 0.001.

**Table 3:** Prediction strength of laboratory tests on successive days using baseline cut-off values.

	Baseline (1st test)	2nd test	3rd test	4th test	5th test	6th test	7th test	8th test
AUC	0.86	0.88	0.87	0.86	0.85	0.83	0.82	0.83
[95% CI]	[0.83, 0.89]	[0.84, 0.91]	[0.83, 0.90]	[0.82, 0.85]	[0.82, 0.88]	[0.80, 0.86]	[0.78, 0.85]	[0.79, 0.85]
C-index	0.85	0.86	0.86	0.84	0.83	0.84	0.81	0.82
[95% CI]	[0.83, 0.86]	[0.83, 0.89]	[0.82, 0.86]	[0.82, 0.86]	[0.80, 0.86]	[0.81, 0.87]	[0.77, 0.83]	[0.79, 0.83]

**Table 4:** Prediction strength of laboratory tests on successive days without using cut-off values.

	Baseline (1st test)	2nd test	3rd test	4th test	5th test	6th test	7th test	8th test
AUC	0.86	0.86	0.87	0.87	0.87	0.87	0.88	0.88
[95% CI]	[0.84, 0.88]	[0.83, 0.89]	[0.85, 0.90]	[0.86, 0.89]	[0.85, 0.90]	[0.85, 0.91]	[0.86, 0.90]	[0.85, 0.91]
C-index	0.85	0.86	0.86	0.86	0.86	0.87	0.87	0.88
[95% CI]	[0.83, 0.89]	[0.83, 0.87]	[0.84, 0.89]	[0.83, 0.89]	[0.84, 0.88]	[0.85, 0.90]	[0.86, 0.90]	[0.86, 0.91]

**Table 5:** Prediction strength of cumulative laboratory tests without using cut-off values.

	Baseline (1st test)	2nd test	3rd test	4th test	5th test	6th test	7th test	8th test
AUC	0.86	0.87	0.87	0.88	0.88	0.88	0.89	0.90
[95% CI]	[0.82, 0.89]	[0.85, 0.89]	[0.85, 0.89]	[0.86, 0.90]	[0.86, 0.90]	[0.86, 0.91]	[0.87, 0.90]	[0.88, 0.92]
C-index	0.85	0.85	0.85	0.86	0.86	0.86	0.87	0.87
[95% CI]	[0.82, 0.89]	[0.82, 0.89]	[0.83, 0.89]	[0.84, 0.89]	[0.83, 0.89]	[0.83, 0.89]	[0.85, 0.89]	[0.85, 0.90]

**Table 6:** Easy-to-use score system for early prediction of severe COVID-19 disease.

Characteristics	Cut-off	Score
**Demographics**		
Male gender	Present	0.65
Age (select highest score)		
[60, 64]	Present	0.46
[65, 69]	Present	0.89
[70, 74]	Present	0.97
≥75	Present	2.3
**Past comorbidities**		
Diabetes mellitus	Present	1.7
Hypertension	Present	2
Atrial fibrillation	Present	1.7
Heart failure	Present	3
Ischemic heart disease	Present	1.6
Peripheral vascular disease	Present	2.7
Stroke	Present	2
Dementia or Alzheimer’s	Present	2.3
Liver diseases	Present	1.7
Gastrointestinal bleeding	Present	1.6
Cancer	Present	1.8
**Complete blood count**		
Neutrophil, ×10^9^/L	>5.43	0.28
Lymphocyte, ×10^9^/L	<1.48	1.5
Platelet, ×10^9^/L	<321	0.0083
Hematocrit, L/L	<0.38	9
**Liver and renal function tests**		
Potassium, mmol/L	>4.35	0.39
Albumin, g/L	<33.8	0.096
Sodium, mmol/L	<135.24	0.18
Urea, mmol/L	>6.1	0.088
Creatinine, μmol/L	>97.2	0.0033
Alanine transaminase, U/L	>25.8	0.0067
Bilirubin, μmol/L	>10.8	0.028
**Lipid and glucose tests**		
Low-density lipoprotein, mmol/L	<1.78	0.74
High-density lipoprotein, mmol/L	<0.9	1.3
Cholesterol, mmol/L	<3.1	0.6
Glucose, mmol/L	<5.55	0.12
**Cardiac, clotting, inflammatory, and acid–base tests**		
D-dimer, ng/mL	>831.5	0.0005
High sensitive troponin-I, ng/L	>9.95	0.0003
Lactate dehydrogenase, U/L	>289	0.0056
APTT, second	>32.8	0.031
Prothrombin time/INR, second	>13	0.024
C-reactive protein, mg/dL	>0.88	0.14
Base excess, mmol/L	<−3.5	0.096

**Fig. 2:**
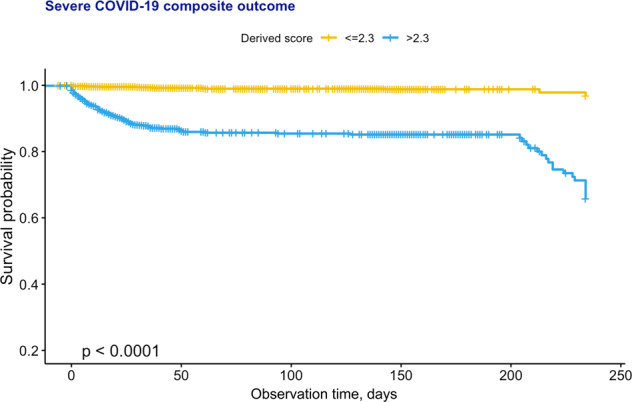
Survival anlysis for the cohort from Hong Kong, China. Survival curve of COVID-19 patients stratified by dichotomized risk score.

Patients meeting the primary outcome (*n* = 212) have significantly higher risk score (median: 5.13, 95% CI: 3.13–7.43, max: 18.6) than those who did not (median: 1.41, 95% CI: 0.65–5.94, max: 18.2) (Table 7), indicating the significant risk stratification performance of the clinical risk score (OR: 17.1, 95% CI: 11–26.6) (Table 8). Survival curves stratified by the dichotomized risk score are shown in Fig. 2, where yellow and blue curves represent the survival analysis for patients with a clinical risk score is larger and smaller than the cut-off, respectively.

**Table 7:** Derived score characteristics of patients with/without composite outcome.

	No. of composite (*n* = 4233)	Composite (*n* = 209)	*P* value
	Median (IQR); max	Median (IQR); max	
Derived risk score	1.41 (0.65–5.94); 18.22	5.13 (3.13–7.43); 18.61	<0.0001***

*p ≤ 0.05, **p ≤ 0.01, ***p ≤ 0.001.

**Table 8:** Stratification performance of score and dichotomized score system.

	Cut-off	OR (95% CI)	*Z* value	*P* value
Score	2.3	1.26 (1.22–1.29)	17.4	<0.0001***
Score ≥ 2.3	–	17.1 (11.0–26.6)	12.6	<0.0001***

**Fig. 3:**
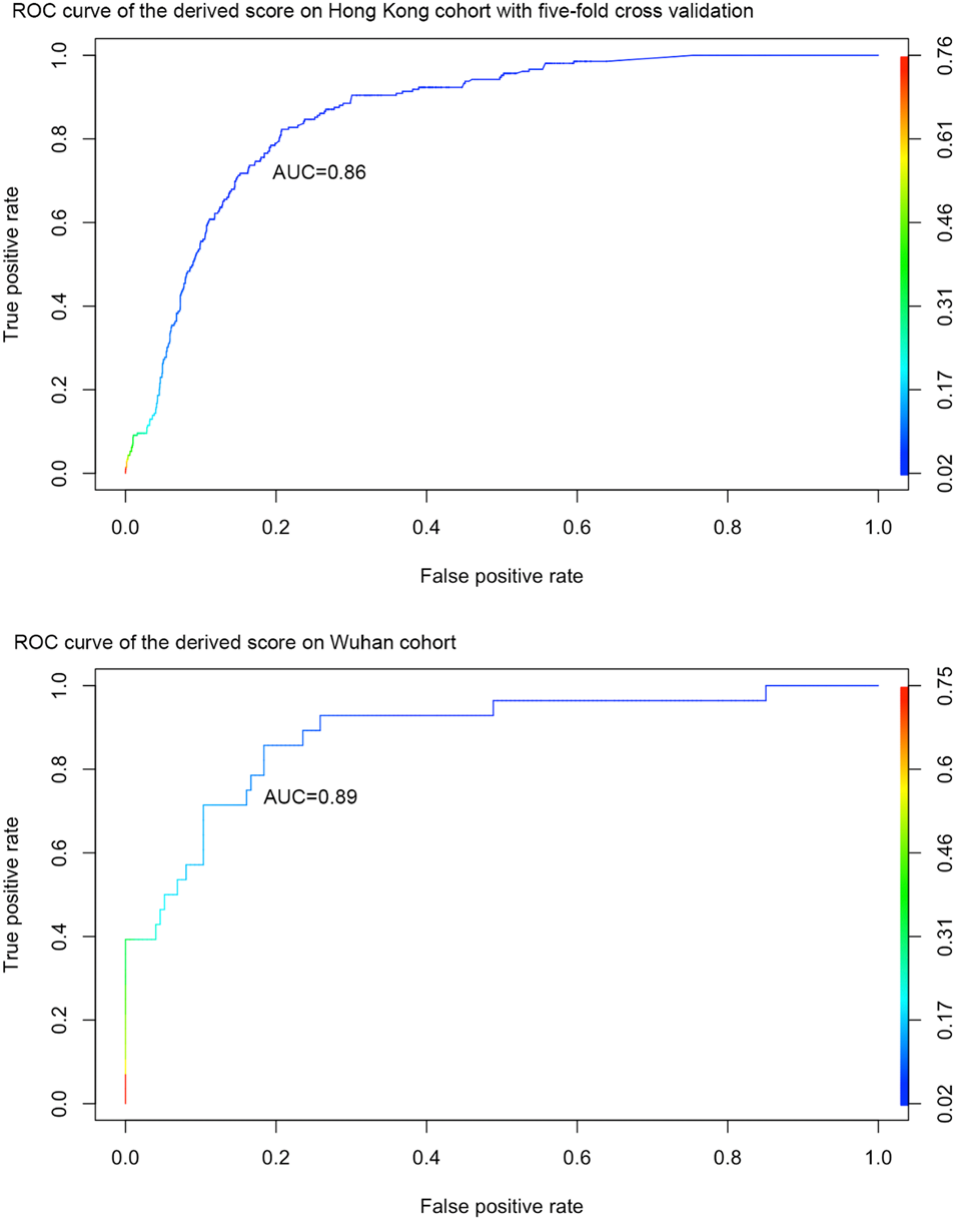
Receiver operating characteristic (ROC) analysis. ROC curves for classifying composite outcome of COVID-19 patients with dichotomized risk score on Hong Kong cohort (fivefold cross-validation) and Wuhan cohort.

For external validation, a total of 202 patients (48% males) from the Wuhan Heart Hospital were included. Comparisons of different performance measures for the clinical risk score for the Hong Kong cohort (fivefold cross-validation) and Wuhan cohort are detailed in Table 9. Receiver operating characteristic curve (ROC) of predicting adverse composite outcome of COVID-19 patients with the dichotomized risk score cut-off is shown in Fig. 3 (Hong Kong cohort: top panel; Wuhan cohort: bottom panel). As the Wuhan cohort did not routinely have AST tested, this variable was excluded for the performance comparisons. The AUC of 0.86 for the Hong Kong cohort (fivefold cross-validation) and 0.89 for the Wuhan cohort.

**Table 9:** Comparisons of different performance measures for the clinical risk score for the Hong Kong cohort (fivefold cross-validation) and the Wuhan cohort.

Cohort	*N*	AUC [95% CI]	Accuracy [95% CI]	Specificity [95% CI]	Precision [95% CI]
Hong Kong	4442	0.86 [0.82, 0.91]	0.83 [0.80, 0.86]	0.85 [0.83, 0.89]	0.85 [0.82, 0.90]
Wuhan	202	0.89 [0.85, 0.93]	0.87 [0.85, 0.91]	0.88 [0.85, 0.92]	0.89 [0.86, 0.90]

## Discussion

In this study, we developed a simple clinical score to predict severe COVID-19 disease based on age, gender, medical comorbidities, medication records, and laboratory examination results. We compared the prediction strengths of different criteria for the clinical risk score for out-of-sample validation for the Hong Kong cohort (fivefold cross-validation) and external validation for the Wuhan cohort, with AUC as 0.86 (95% CI: 0.82–0.91) and 0.89 [0.85–0.93], respectively. The derived score system achieved good predictions even without the consideration of clinical parameters such as symptoms, blood pressure, oxygen status on presentation, or chest radiograph results.

COVID-19 disease has placed significant pressures on healthcare systems worldwide. Early risk stratification may better direct the use of limited resources and allow clinicians to triage patients and make clinical decisions based on limited evidence objectively. For example, low-risk patients may require simple monitoring only, while patients that are likely to deteriorate may benefit from intensive drug treatment or intensive care. Currently, the availability of simple clinical risk scores for risk stratification is limited. The COVID-GRAM predicts development of critical illness, based on symptoms, radiograph results, clinical and laboratory details^24^. Similarly, the 4C Mortality Score included eight variables readily available at initial hospital assessment: age, sex, number of comorbidities, respiratory rate, peripheral oxygen saturation, level of consciousness, urea level, and C-reactive protein (score range 0–21 points)^25^. These scores produced moderately accurate predictions with C-index values of 0.86 and 0.61–0.76, respectively. A systematic review and meta-analysis have recently summarized different risk scores that have been developed by investigators from different countries^26^. As reported, the most frequently reported predictors were age, clinical status such as temperature, imaging results from chest radiography, and lymphocyte count. Recently, a study including 3927 patients from 33 hospitals developed the COVID-19 Mortality Risk (CMR) tool using the XGBoost algorithm^27^. This score is based on age, blood urea nitrogen, CRP, creatinine, glucose, AST, and platelet counts. Different teams in our country have already used a data-driven approach to develop predictive risk models for COVID-19 to predict viral transmission^28,29^, adverse outcomes^30,31^ and even to determine effects of risk perceptions on behaviors in response to the outbreak^32^. For example, our team recently developed a risk model based on non-linear interactions between different variables to predict intensive care unit admission using a tree-based machine learning model^30^. The above models are based on individual-level patient data. Where these are not available, investigators have successfully developed a useful model by using aggregate epidemiological reports of COVID-19 case fatality events^33^.

In this study, with an expanded cohort, we developed a simple and easy-to-use model was based on past comorbidity and laboratory data only, without needing clinical assessment details or chest imaging interpretation. The model based on test results taken on the day of admission already demonstrated an excellent predictive value with a *C*-statistic of 0.89. Incorporation of test results on successive time points did not further improve risk prediction, indicating that initial data are sufficient to produce accurate predictions of severe disease. Our model can aid clinical decision making as early intervention may be associated with better outcomes^19–23^.

The major limitation of this study is that it is based on a territory-wide cohort from a single city in China (Hong Kong). However, the risk score was independently validated using an external cohort from another city (Wuhan). We recognize that the baseline demographic and clinical characteristics of COVID-19 patients may differ in other countries. The model should be further externally validated using patient data involving from other geographical regions to allow further generalization.

In conclusion, simple clinical score based on only demographics, comorbidities, medication records, and laboratory tests accurately predicted severe COVID-19 disease, even without including symptoms on presentation, blood pressure, oxygen status, or chest radiograph results. The model based on test results taken on the day of admission showed an excellent predictive value. Incorporation of test results on successive time points did not further improve risk prediction. Both out-of-sample fivefold cross-validation on Hong Kong cohort and independent external validation on Wuhan cohort demonstrated the significant risk stratification performance of the derived score system for severe COVID-19 disease. The presented score system tool used commonly available clinical and laboratory results and does not require imaging results or advanced testing, and therefore can be particularly useful in facilities with constrained resources or remote hospitals with limited diagnostic capabilities such as computed tomography scans.

## Methods

### Study design and population

This study was approved by the Institutional Review Board of the University of Hong Kong/Hospital Authority Hong Kong West Cluster. The need for informed consent was waived by the Ethics Committee owing to the retrospective and observational nature of this study. This was a retrospective, territory-wide cohort study of patients undergoing COVID-19 RT-PCR testing between 1 January 2020 and 22 August 2020 in Hong Kong, China. The patients were identified from the Clinical Data Analysis and Reporting System (CDARS), a territory-wide database that centralizes patient information from 43 local hospitals and their associated ambulatory and outpatient facilities to establish comprehensive medical data, including clinical characteristics, disease diagnosis, laboratory results, and drug treatment details. The system has been previously used by both our team and other teams in Hong Kong^34,35^, including recently COVID-19 research^36,37^. This system captures PCR tests performed in Accident and Emergency, outpatient and inpatient settings. Patients demographics, prior comorbidities, hospitalization characteristics before admission due to COVID-19, medication prescriptions, laboratory examinations of complete blood counts, biochemical tests, diabetes mellitus tests, cardiac function tests, c-reactive protein, and blood gas tests were extracted. The list of ICD-9 codes for comorbidities and intubation procedures are detailed in the Supplementary Tables 1 and 2.

### Outcomes and statistical analysis

The primary outcome was a composite of need for intensive care admission, intubation, or all-cause mortality with follow-up until 8 September 2020. Mortality data were obtained from the Hong Kong Death Registry, a population-based official government registry with the registered death records of all Hong Kong citizens linked to CDARS. Patients who passed away 30 days later or longer after discharge were excluded. The need for ICU admission and intubation were extracted directly from CDARS. There was no adjudication of the outcomes as this relied on the ICD-9 coding or a record in the death registry. However, the coding was performed by the clinicians or administrative staff, who were not involved in the mode development. Descriptive statistics are used to summarize baseline clinical characteristics of all patients with COVID-19 and based on the occurrence of the primary outcome. Continuous variables were presented as median (95% confidence interval [CI] or interquartile range [IQR]) and categorical variables were presented as count (%). The Mann–Whitney *U* test was used to compare continuous variables. The *χ*^2^ test with Yates’ correction was used for 2 × 2 contingency data. Univariate logistic regression identifies significant mortality risk predictors. Odds ratios (ORs) with corresponding 95% CIs and *P* values were reported. There was no imputation performed for missing data. An easy-for-use predictive model was developed using the beta coefficients for different predictors identified from logistic regression. Successive laboratory tests at least 24 h apart were used. No blinding was performed for the predictor as the values were obtained from the electronic health records automatically.

### Development of different scoring systems

Three different models were developed.

Model 1: optimum cut-off values of different variables at baseline were obtained from receiver operating characteristic (ROC) analysis. Laboratory examinations on for each successive 24 h was compared to cut-off to determine whether the criterion was met at each time point.

Model 2: the criterion was met if the value was abnormal by standard laboratory criteria, without consideration of optimal cut-off values.

Model 3: laboratory test results are compared to the criteria without cut-off values, to determine if they were met on successive testing. For example, if a particular criterion is met on day 1, then they will automatically fulfill the criteria for subsequent days.

A simple and easy-to-use score system was built based on beta coefficients using logistic regression analysis. The risk score of each COVID-19 patient was then calculated. The derived score system was evaluated within-sample fivefold cross testing set and out-of-sample dataset from Wuhan for external validation. The model was not recalibrated after validation.

### External validation

For external validation, patients admitted to the Wuhan Asia General Hospital^38^, Wuhan, China, between 10 February and 10 March 2020, were included. Diagnosis of COVID-19 was based on positive PCR test and ground glass shadows in the lungs on computed tomography scan, with follow-up 2 weeks post-discharge. Lipid and aspartate aminotransferase were not routinely collected and therefore not included for validation.

### Performance of the score

Performance of the score system was evaluated based on its ability to discriminate the composite outcome for each population. The results for in-sample testing set, and for external out-of-sample validation cohort were reported, with the corresponding CIs. The area under the curve (AUC), accuracy, specificity, and precision were computed for all patient subpopulations. Receiver operating characteristic (ROC) curves were created for each of the cohorts with the derive score system to predict the adverse composite outcome. All statistical tests were two-tailed and considered significant if *p* value < 0.001. They were performed using RStudio software (Version: 1.1.456) and Python (Version: 3.6).

### Reporting summary

Further information on experimental design is available in the Nature Research Reporting Summary linked to this paper.

## Supplementary information

Supplementary Information

Reporting Summary

## Data Availability

The data that support the findings of this study are available from the corresponding author upon reasonable request.
