# Supplementary material for: Development of a multivariable prediction model for severe COVID-19 disease: a population-based study from Hong Kong
Source: NPJ Digit Med. 2021 Apr 8;4:66. doi: 10.1038/s41746-021-00433-4 (PMC8032826; doi:10.1038/s41746-021-00433-4)
Supplement: Supplementary file 1 — Supplementary Information [file 41746_2021_433_MOESM1_ESM.pdf]

Supplementary Appendix

Supplementary Table 1. Codes for Comorbidities of COVID-19 Patients

Diabetes mellitus 250 250.01 250.02 250.03 250.1 250.11 250.12 250.13 250.2 250.21 250.22 250.23 250.3 250.31 250.32 250.33 250.4 250.41 250.42 250.43 250.5 250.51 250.52 250.53 250.6 250.61 250.62 250.63 250.7 250.71 250.72 250.73 250.8 250.81 250.82 250.83 250.9 250.91 250.92 250.93
Hypertension 401 401.1 401.9 402 402.01 402.1 402.11 402.9 402.91 403 403.01 403.1 403.11 403.9 403.91 404 404.01 404.02 404.03 404.1 404.11 404.12 404.13 404.9 404.91 404.92 404.93 405 405.01 405.09 405.1 405.11 405.19 405.9 405.91 405.99 437.2
Heart failure 428 428 428.1 428.2 428.2 428.21 428.22 428.23 428.3 428.3 428.31 428.32 428.33 428.4 428.4 428.41 428.42 428.43 428.9 398.91 402.01 402.11 402.91 404.01 404.03 404.11 404.13 404.91 404.93
Atrial fibrillation 427.31 429.4
Liver diseases 456 456.1 456.2 572.2 572.3 572.4 572.8 571.4 571.5 571.6
Dementia and Alzheimer 331.82 290 290.1 290.11 290.12 290.13 290.2 290.21 290.3 290.4 290.41 290.42 290.43 290.8 290.9 294.2 294.1 294.11 294.21 332 46.1 333.4 340 42331 331.19 294.29
COPD 490 491 492 493 494 495 496 491.1 491.2 491.21 491.22 491.8 491.9 492.8 493.01 493.02 493.1 493.11 493.12 493.2 493.21 493.22 493.8 493.81 493.82 493.9 493.91 493.92 494.1 495.1 495.2 495.3 495.4 495.5 495.6 495.7 495.8 495.9
Peripheral vascular disease 250.7 443.9 443 443.1 443.2 443.21 443.22 443.23 443.24 443.29 443.8 443.81 443.82 443.89 441 443.9 785.4 V43.4
Stroke 435 435.1 435.2 435.3 435.8 435.9 433.81 433.91 434 436 437 437.1 433.31 433.01 434.01 434.1 434.11 434.9 434.91 437.2 437.3 437.4 437.5 437.6 437.7 437.8 437.9
Gastrointestinal bleeding 531 531.2 531.4 531.6 532 532.2 532.4 532.6 533 533.2 533.4 533.6 534 534.2 534.4 534.6 535.01 535.11 535.21 535.31 535.41 535.51 535.61 535.71 562.02 562.03 562.12 562.13 569.3 569.85 569.86 578 578.1 578.9
Ischemic heart disease 410.01 410.02 410.1 410.11 410.12 410.2 410.21 410.22 410.3 410.31 410.32 410.4 410.41 410.42 410.5 410.51 410.52 410.6 410.61 410.62 410.7 410.71 410.72 410.8 410.81 410.82 410.9 410.91 410.92 411 411.1 411.8 411.81 411.89 413 413.1 413.9 414 414.01 414.02 414.03 414.04 414.05 414.06 414.07 414.1 414.11 414.12 414.19 414.2 414.3 414.4 414.8 414.9 410 412
Cancer 140-239

Supplementary Table 2. Codes for Identifying Patients Needing Intubation

Procedure	Codes and Description
Intubation	Cont invasive mech vent->96 hours (96.72:0)
	Cont invasive mech vent-<96 hours (96.71:0)
	Invasive mechanical ventilation (96.70:0)
	Endotracheal intubation (96.04:0)
	Respiratory tract intubation (96.05:0)

Supplementary Table 3. Baseline clinical characteristics of male and female COVID-19 patients.

* for p≤ 0.05, ** for p ≤ 0.01, *** for p ≤ 0.001.

**Characteristics**	**Male (N=2227) Median (IQR);Max;N or Count(%)**	**Female (N=2215) Median (IQR);Max;N or Count(%)**	**P value**
Outcomes			
Composite	138(6.19%)	71(3.20%)	<0.0001***
Mortality	57(2.55%)	36(1.62%)	0.0429*
ICU	69(3.09%)	27(1.21%)	<0.0001***
Intubation	68(3.05%)	30(1.35%)	0.0002***
**Demographics**			
Age	44.1(27.9-61.4);95.6;n=2227	45.22(30.1-59.86);100.6;n=2215	0.7033
[60,64]	213(9.56%)	188(8.48%)	0.275
[65,69]	164(7.36%)	125(5.64%)	0.0342*
[70,74]	103(4.62%)	91(4.10%)	0.4635
>=75	140(6.28%)	142(6.41%)	0.9219
Past comorbidities			
Charlson score	1.0(1.0-2.0);13.0;n=2227	1.0(1.0-2.0);12.0;n=2215	0.2812
Diabetes mellitus	37(1.66%)	37(1.67%)	0.925
Hypertension	301(13.51%)	300(13.54%)	0.9844
Heart failure	1(0.04%)	6(0.27%)	0.1292
Congestive heart failure	1(0.04%)	4(0.18%)	0.3684
Atrial fibrillation	27(1.21%)	16(0.72%)	0.134
Liver diseases	3(0.13%)	4(0.18%)	0.9948
Dementia and Alzheimer	3(0.13%)	5(0.22%)	0.7187
AMI	25(1.12%)	5(0.22%)	0.0006***
COPD	19(0.85%)	15(0.67%)	0.6202
IHD	77(3.45%)	33(1.48%)	<0.0001***
PVD	4(0.17%)	3(0.13%)	0.9938
Stroke	38(1.70%)	32(1.44%)	0.5696
Gastrointestinal bleeding	42(1.88%)	29(1.30%)	0.165
Cancer	40(1.79%)	55(2.48%)	0.1484
Obesity	1(0.04%)	5(0.22%)	0.2187
Medications			
ACEI	97(4.35%)	63(2.84%)	0.0115*
ARB	73(3.27%)	76(3.43%)	0.8486
Steroid	125(5.61%)	133(6.00%)	0.6438
Kaletra	327(14.68%)	344(15.53%)	0.5251
Ribavirin	281(12.61%)	246(11.10%)	0.1813
Interferon beta-1B	358(16.07%)	358(16.16%)	0.9786

Hydroxychloroquine	13(0.58%)	15(0.67%)	0.8403
Calcium channel blockers	244(10.95%)	233(10.51%)	0.7081
Beta blockers	101(4.53%)	104(4.69%)	0.864
Diuretics for hypertension	25(1.12%)	29(1.30%)	0.6717
Nitrates	40(1.79%)	22(0.99%)	0.0339*
Antihypertensive drugs	74(3.32%)	18(0.81%)	<0.0001***
Antidiabetic drugs	140(6.28%)	96(4.33%)	0.0073**
Statins and fibrates	229(10.28%)	161(7.26%)	0.0014**
Lipid-lowering drugs	221(9.92%)	158(7.13%)	0.0027**
Anticoagulants	96(4.31%)	61(2.75%)	0.0085**
Antiplatelets	108(4.84%)	82(3.70%)	0.0824
Complete blood count			
Mean corpuscular volume, fL	86.8(83.1-90.0);105.9;n=1156	86.87(82.7-90.2);110.6;n=1235	0.9588
Basophil, x10^9/L	0.01(0.0-0.02);0.2;n=1444	0.01(0.0-0.02);0.14;n=1475	0.1596
Eosinophil, x10^9/L	0.04(0.0-0.1);1.0;n=1498	0.02(0.0-0.1);3.53;n=1539	<0.0001***
Lymphocyte, x10^9/L	1.3(0.9-1.8);16.99;n=1503	1.4(1.0-1.86);13.7;n=1542	0.0033**
Lymphocyte binary	883.0(39.64%)	843.0(38.05%)	0.488
Blast, x10^9/L	0.0(0.0-0.0);0.1;n=60	0.0(0.0-0.0);0.2;n=76	0.6632
Metamyelocyte, x10^9/L	0.16(0.08-0.24);0.7;n=10	0.08(0.06-0.1);0.12;n=4	0.2283
Monocyte, x10^9/L	0.5(0.39-0.67);2.32;n=1503	0.45(0.33-0.6);3.15;n=1542	<0.0001***
Neutrophil, x10^9/L	3.32(2.44-4.49);18.63;n=1503	3.19(2.3-4.3);17.37;n=1542	0.0022**
Neutrophil binary	191.0(8.57%)	182.0(8.21%)	0.7312
White blood bount, x10^9/L	5.5(4.4-6.83);23.62;n=1532	5.2(4.14-6.6);23.9;n=1570	0.0002***
Mean cell haemoglobin, pg	30.1(28.7-31.39);36.5;n=1533	29.7(28.3-31.1);37.0;n=1569	<0.0001***
Myelocyte, x10^9/L	0.28(0.07-0.36);1.29;n=19	0.21(0.11-0.32);0.62;n=11	0.88
Platelet, x10^9/L	206.0(168.0-257.0);778.0;n=1533	222.0(183.0-280.0);722.0;n=1569	<0.0001***
Platelet binary	1406.0(63.13%)	1356.0(61.21%)	0.541
Reticulocyte, x10^9/L	42.6(38.57-71.49);87.7;n=7	30.2(29.2-62.4);318.0;n=5	0.7453
Red blood count, x10^12/L	4.93(4.61-5.3);7.45;n=1533	4.44(4.16-4.75);6.57;n=1570	<0.0001***
Hematocrit, L/L	0.42(0.39-0.44);0.516;n=264	0.38(0.36-0.4);0.5;n=244	<0.0001***

Liver and renal function tests			
K/Potassium, mmol/L	3.9(3.6-4.12);6.96;n=1141	3.75(3.5-4.0);7.7;n=1148	<0.0001***
Urate, mmol/L	0.33(0.28-0.44);0.635;n=30	0.25(0.22-0.28);0.55;n=21	0.0037**
Albumin, g/L	41.0(37.72-44.65);118.2;n=1149	40.5(37.0-43.6);201.0;n=1153	0.0003***
Na/Sodium, mmol/L	139.0(136.8-140.0);146.2;n=1144	139.0(137.5-141.0);147.1;n=1151	<0.0001***
Urea, mmol/L	4.34(3.6-5.3);59.3;n=1143	3.5(2.8-4.3);31.64;n=1151	<0.0001***
Protein, g/L	74.0(70.0-77.25);92.0;n=1006	74.0(70.85-78.0);92.7;n=1028	0.0979
Creatinine, umol/L	82.0(73.0-92.0);1248.0;n=1152	60.0(54.0-68.0);1280.0;n=1152	<0.0001***
Alkaline phosphatase, U/L	68.2(56.05-83.55);550.0;n=1142	63.0(52.0-78.0);540.0;n=1150	<0.0001***
Aspartate transaminase, U/L	30.0(22.0-46.65);1713.0;n=336	25.0(20.0-37.5);863.0;n=308	0.0001***
Alanine transaminase, U/L	27.0(19.0-41.0);320.0;n=912	20.0(14.7-29.0);902.0;n=906	<0.0001***
Bilirubin, umol/L	8.6(6.2-12.0);60.4;n=1142	6.4(4.7-9.0);148.4;n=1149	<0.0001***
Lipid and glucose tests			
Triglyceride, mmol/L	1.44(1.03-2.1);9.35;n=175	1.32(0.99-1.97);7.499;n=115	0.2987
Low-density lipoprotein, mmol/L	2.49(1.93-3.1);6.8719;n=157	2.36(1.9-3.14);5.8679;n=102	0.5957
High-density lipoprotein, mmol/L	1.02(0.82-1.24);2.1;n=162	1.17(0.96-1.45);2.97;n=106	0.0002***
Cholesterol, mmol/L	4.4(3.6-5.01);9.4;n=163	4.3(3.8-5.16);8.5;n=109	0.2792
HbA1c, g/dL	14.6(13.6-15.4);94.1;n=1541	13.0(12.0-13.7);84.0;n=1576	<0.0001***
Glucose, mmol/L	5.7(5.0-6.92);22.5;n=1116	5.6(5.06-6.84);32.1;n=1087	0.4946
Cardiac, clotting, inflammatory, and acid-base tests			
D-dimer, ng/mL	410.0(241.8-756.95);10000.0;n=312	360.2(236.5-581.1);6579.9;n=276	0.0315*
High sensitive troponin-I, ng/L	3.7(1.9-7.96);12827.6;n=727	3.0(1.12-4.43);203.2;n=659	<0.0001***
Lactate dehydrogenase, U/L	198.0(167.0-254.0);1116.0;n=1269	195.0(164.0-235.0);751.0;n=1203	0.0088**
APTT, second	31.1(27.8-34.9);120.0;n=782	30.3(27.2-33.8);57.8;n=811	0.0008***
Prothrombin time/INR, second	12.1(11.6-12.7);110.0;n=560	11.9(11.4-12.5);28.5;n=550	<0.0001***

C-reactive protein, mg/dL	0.44(0.14-1.76);33.99;n=1552	0.31(0.11-1.11);28.1;n=1556	<0.0001***
HCO3	22.9(19.95-24.45);32.8;n=95	23.6(20.45-25.95);31.0;n=71	0.1911
Base Excess	-0.6(-2.85-1.4);7.2;n=310	-0.4(-2.0-1.8);9.5;n=200	0.1436
Bicarbonate	24.4(21.5-26.2);32.1;n=159	24.6(22.6-27.1);33.2;n=95	0.1377
Blood pCO2	4.8(4.0-5.62);10.9;n=311	4.96(4.3-5.8);10.9;n=200	0.1039
Blood pH	7.4(7.4-7.5);7.6;n=311	7.4(7.4-7.5);7.6;n=200	0.408
Calcium, mmol/L	1.12(1.1-1.16);1.33;n=22	1.14(1.06-1.18);1.21;n=14	0.9094

COPD: chronic obstructive pulmonary disease; ACEI: angiotensinogen converting enzyme inhibitor; ARB: angiotensin receptor blocker; APTT: activated partial thromboplastin time.
